# Identifying Diabetic Retinopathy in the Human Eye: A Hybrid Approach Based on a Computer-Aided Diagnosis System Combined with Deep Learning

**DOI:** 10.3390/tomography10020017

**Published:** 2024-02-05

**Authors:** Şükran Yaman Atcı, Ali Güneş, Metin Zontul, Zafer Arslan

**Affiliations:** 1 Department of Computer Engineering, İstanbul Aydın University, Istanbul 34295, Turkey; aligunes@aydin.edu.tr (A.G.); zaferaslan@aydin.edu.tr (Z.A.); 2 Department of Computer Engineering, Sivas University of Science and Technology, Sivas 58140, Turkey; metinzontul@sivas.edu.tr

**Keywords:** diabetic retinopathy, image classification, object detection, computer-aided diagnosis, convolutional neural network (CNN)

## Abstract

Diagnosing and screening for diabetic retinopathy is a well-known issue in the biomedical field. A component of computer-aided diagnosis that has advanced significantly over the past few years as a result of the development and effectiveness of deep learning is the use of medical imagery from a patient’s eye to identify the damage caused to blood vessels. Issues with unbalanced datasets, incorrect annotations, a lack of sample images, and improper performance evaluation measures have negatively impacted the performance of deep learning models. Using three benchmark datasets of diabetic retinopathy, we conducted a detailed comparison study comparing various state-of-the-art approaches to address the effect caused by class imbalance, with precision scores of 93%, 89%, 81%, 76%, and 96%, respectively, for normal, mild, moderate, severe, and DR phases. The analyses of the hybrid modeling, including CNN analysis and SHAP model derivation results, are compared at the end of the paper, and ideal hybrid modeling strategies for deep learning classification models for automated DR detection are identified.

## 1. Introduction

Diabetes, a widely recognized chronic condition, has seen a concerning surge in global fatalities. Its adverse effects manifest across various parts of the human body, notably impacting the retinas of the eyes, often resulting in significant vision impairment during advanced stages of diabetic disease. Diabetic retinopathy (DR) is a condition wherein diabetes affects the eyes [1,2], primarily due to elevated blood sugar levels affecting the retina’s blood vessels. Recent studies project that, by 2040, diabetes will affect approximately 642 million adults worldwide, a significant increase from 2012 to 2021 [3,4], with an estimated one in three individuals with diabetes experiencing DR [5]. Various levels of DR have been categorized, including stage zero, indicating no retinopathy.

On the contrary, stages one, two, and three correspond to mild, moderate, and severe non-proliferative DR conditions. However, the onset of proliferative DR is associated with stage four, a critical and substantial phase [6]. This stage poses a severe risk, potentially leading to various vision-related issues like blurriness or even blindness, significantly impacting patients’ overall health and daily life routines. Therefore, the early detection of DR is crucial in averting potentially fatal outcomes for patients [7]. DR diagnosis encounters multifaceted challenges, and current methods bear several limitations. Early detection remains a foremost hurdle as the condition often manifests asymptomatically in its initial stages, delaying timely intervention. Accessibility to screening facilities, particularly in remote areas, poses another obstacle, impeding regular examinations for diabetic individuals in underserved communities. Moreover, the subjectivity inherent in diagnostic approaches, which are reliant on individual interpretation by ophthalmologists or trained graders, introduces variability and inconsistency in diagnoses. The cost-intensive nature of certain diagnostic procedures, such as fluorescein angiography, coupled with the requirement for specialized equipment and expertise, limits their widespread application and affordability, especially in resource-constrained settings. Compared to manual, time-consuming screening, automatic computer-aided diagnosis screening for DR can accelerate the process much more straightforwardly [8]. Patient compliance with follow-up appointments also presents a challenge, influenced by factors like awareness, convenience, and financial constraints.

Additionally, integrating diverse patient data for comprehensive analysis remains complex, and scaling current methodologies to accommodate burgeoning diabetic populations poses significant logistical hurdles. Addressing these challenges necessitates innovative approaches leveraging technologies such as artificial intelligence and telemedicine to improve accessibility, accuracy, and objectivity in DR diagnosis while streamlining data management and enhancing scalability for broader population screening. Recent advancements in computing hardware have made it incredibly simple to use machine learning techniques widely, which has been extremely advantageous for the biomedical field [4]. As a result, compared to traditional methods, the application of deep learning has significantly improved the diagnosis of DR [9].

The primary objective of this study is to conduct a comprehensive experimental comparison using computer vision techniques to determine the most effective pre-trained neural network for classifying, identifying objects, and segmenting multi-class imbalanced datasets related to DR. Imbalanced datasets are characterized by a significant disparity in population between majority and minority classes, leading to skewed classification outcomes biased toward the majority classes [10,11,12]. Unlike many real-world biomedical datasets, those associated with DR exhibit a highly skewed distribution among classes. This poses challenges in developing learning models for multi-class imbalanced datasets, as it is difficult to anticipate which classes will dominate or be underrepresented in each distribution [13,14,15]. Additionally, selecting the appropriate assessment measures to gauge how well various learning models perform in light of their respective computer tasks is another challenge. In contrast to the binary class problem, the distribution of samples across many classes is more complicated. Current computer-aided systems (CADs) for DR detection through image processing encounter notable limitations stemming from the complexity and variability of retinal images. These systems often struggle with detecting subtle or early-stage manifestations of the disease due to variations in image quality, lighting conditions, and artifacts [14].

The robustness and accuracy of these systems can be compromised when handling images with irregularities or when confronted with diverse datasets. Additionally, while these systems leverage sophisticated algorithms, they might need more adaptability to be effectively generalized across different populations or clinical settings, potentially leading to limited diagnostic accuracy in specific scenarios. The reliance on curated datasets might restrict the system’s ability to encounter and learn from the diverse spectrum of real-world clinical variations. Overcoming these limitations requires the continuous refinement of image-processing algorithms, robustness testing across varied image datasets, and the development of adaptable models capable of accommodating the diverse nature of retinal images encountered in clinical practice. Effective models are required to reliably diagnose diabetes and determine type of diabetes based on automated diagnosis employing classification tasks. Moreover, it is crucial to find anomalies in retinal images utilizing object detection and segmentation techniques so that the patient with diabetes can receive accurate therapy early on and reduce their risk of profound vision loss [16].

Classification is regarded as a global labeling task where a single image is assigned a single class label. Object detection is regarded as a sparse labeling task since only a few pixels of the image are labeled. Convolutional neural networks (CNNs), which are widely employed in a variety of application domains, have helped most problems in the computer vision field today expand their resolution capability. The performance of CNNs surpasses that of conventional methods because they can perform automatic feature extraction and classification in a single step [17]. Han et al. [18] divided CNN training into two categories: training from scratch and leveraging pre-trained networks to train models on a target dataset. The models that have already been trained on a larger dataset made up of millions of images are known as pre-trained models.

This study introduces a hybrid deep learning ensemble system designed for the automated diagnosis of different stages of DR. To capture prominent features from multiple retinal lesions depicted in fundus images, we pioneer the use of transfer learning. This novel approach involves amalgamating weights from various models into a unified model, thereby harnessing a diverse array of features and characteristics previously unexplored in this context. In a pioneering methodology unique to this study, a cloned model effectively harnesses averaged weights derived from the training phase. This innovative approach generates distinct features, which are subsequently channeled into a bespoke classifier tailored for evaluating the severity levels of DR. Additionally, we use SHapley Additive exPlanations (SHAP) analysis [19,20] approaches to examine the characteristics that contributed to the results of our prediction models. Each SHAP value defines how much each feature (the region of an image) contributes positively and negatively to the goal prediction in the context of image processing and general ML progress. In a pioneering approach, this study leverages the SHAP methodology, which calculates the local feature importance for each image within the dataset. Unlike existing feature-analysis methods that gauge the extent of features, SHAP assigns a significance value to each feature for a specific prediction. This innovative methodology aims to address the inconsistencies prevalent in current feature importance strategies, mitigating the potential misunderstandings arising from these inconsistencies [14] with synthetics [21]. Notably, the fundus images in this research underwent SHAP analysis, marking a novel utilization of this technique to identify areas manifesting DR’s presence or various stages, delineating the severity of the disease.

Our model is trained to utilize an advanced cyclical learning rates technique [22], employing an automatic learning rate finder to optimize the learning rate decay process, and thereby enhancing model accuracy when handling retinal fundus images depicting varying grades of DR. To facilitate the training and validation of our fusion model, we initiated the process with a widely accessible Kaggle dataset comprising 5590 retinal images categorized into five distinct DR gradations. Two other publicly accessible datasets from the retinal fundus have also been used to evaluate the effectiveness of the suggested ensemble model. Exploring a diverse array of datasets simplifies the assessment of our model’s performance and underscores its reliability in practical scenarios for accurately determining the severity of DR. Our algorithm provides an intelligible way of determining DR severity levels in the context of potential health benefits from a limited dataset by extracting pertinent elements. Even though it is crude and straightforward, our method of DR lesion detection may help professionals diagnose DR effectively. Finally, our suggested strategy was able to achieve good performance for DR detection and lesion extraction by treating a retinal image dataset simply and developing a foundational model. This study introduces a novel method for grading DR severity and extracting DR lesions from retinal images, potentially offering a viable tool for ophthalmologists’ clinical assessments.

## 2. Background of the Hybrid Modelling Literature

The most significant diabetes consequence brought on by an increase in blood glucose levels is DR, and blindness may occur suddenly due to retinal damage from DR. For DR diagnosis, hand-engineered characteristics are frequently combined with conventional machine learning (ML) techniques [23,24,25,26,27]. Many surveys have been examined by Zhu et al. [28] to review conservative approaches. Such methodologies include mathematical morphology, retinal lesion tracking, thresholding with deformable models, clustering-based models, matched filtering models, and hybrid approaches [23,24,29]. Deep learning algorithms have demonstrated outstanding performance in numerous computer vision tasks and have decisively defeated traditional hand-engineered methods in recent years due to the availability of large datasets and incredible computing power. ML models can be adapted to be used in a variety of biomedical domains, and the field has made numerous strides in recent years. Musculoskeletal rehabilitation and rectal, breast, cervical, retinal, and lung cancer treatments are only a few of the significant fields where deep learning-based architectures have benefited biological applications. In this study, our focus is exclusively on employing architectures rooted in ML for the tasks of classification, segmentation, and object recognition. Algorithms based on ML have been created to address diverse tasks related to evaluating retinal fundus images for DR and to develop automated computer-aided diagnosis systems for DR using these deep learning-based approaches.

For DR detection, manually created features are commonly combined with traditional machine learning (ML) methods. These conventional procedures have been the subject of numerous studies [30,31,32,33,34,35]. Mathematical morphology, tracking retinal lesions, thresholding and deformable models, clustering-based models, matched filtering models, and hybrid methods are a few examples of such methodologies. Zhu et al. [28] categorized DR diagnosis based on the adopted approaches, while Arcadu et al. [36] examined algorithms extracting lesion features from fundus scans encompassing blood vessel area, secretions, hemorrhages, microaneurysms, and texture. Fleming et al. [37] evaluated the early research on exudate detection, in which Ting et al. [32,33] offered a summary of retinal vascular segmentation algorithms. Abràmoff et al. [30] and Patton et al. [22] studied plentiful methods for segmenting the optic disc and diagnosing glaucoma. For hand-engineered features, specialized knowledge is required, and choosing the appropriate features requires a thorough examination of all feasible options and time-consuming parameter settings. Methods that rely on manually created features are also not very generalizable. Arcadu et al. [36] used several morphology and segmentation techniques to detect blood vessels, hard exudates, and micro-aneurysms after considering the importance of blood vessel segmentation for effective DR. They studied the wavelet transform to extract the features, which was followed by PCA-based feature selection. For two-class classification, the authors proposed a neural network based on back-propagation. Similarly, in a study by Shanthi and Sabeenian [38], DR was identified using a multilayer perception neural network. A graph cuts technique was used to segment the optic disk as part of an established automatic exudate detection method.

In particular, most artificial neural network-based techniques do not address the overfitting concerns for large-scale fundus images. Raman et al. [39] used a five-class categorization system: mild, moderate, severe, proliferative DR (PDR), and non PDR (NPDR) with engaged optic disc identification for exudates and micro-aneurysm extraction-based DR. In addition, a genetic method was employed to pinpoint exudates. The intersection of aberrant blood vessel thickness was used by Junior and Welfer [40] to specify exudates and other lesions in fundus images. The same techniques were used by Arcadu et al. [36] for DR, including k-means clustering and fuzzy inference systems. The need to establish an autonomous CAD system for DR anomaly detection and severity evaluation was highlighted by Shanthi and Sabeenian [38]. After this improvement, a general CAD model with the ability to detect retinal micro-aneurysms and exudates as well as classify DR data using classifiers such as support vector (SVM) or k-nearest neighbor was employed in fundamental studies [41,42]. Especially in Zhang et al. [43], feature extraction based on discrete wavelet transform was performed to locate DR illnesses in fundus images. Different studies utilized the morphology and texture analysis approach to identify DR characteristics in colored fundus images, including blood vessels and hard exudates. The accuracy of DR classification was identified by Gardner et al. [44] using a neural network of pixel intensity values, and an intense model valuation of more than 80% estimation for a five-class DR level classification was developed.

The objective of this study is to create an effective automated computer-aided design (CAD) system for DR. Several authors have examined several retinal image-processing methods and their applications for CAD-based DR screening in previous studies [45,46]. Studies [31,32,33,41] highlighted image pre-processing, the segmentation of basic and abnormal retinal features, and DR detection methods. Mainly, Patton et al. [45] presented a review on image registration, pre-processing, the segmentation of pathological features, and different imaging modalities for DR diagnosis. Nevertheless, none of the existing studies conducted a comparison and review of contemporary state-of-the-art ML algorithms while incorporating the design of the human eye as a spherical shape. This distinctive aspect sets our study apart, as it illustrates DR points in multiple dimensions.

## 3. Dataset and Methodology

### 3.1. DR Dataset

**Figure 1:**
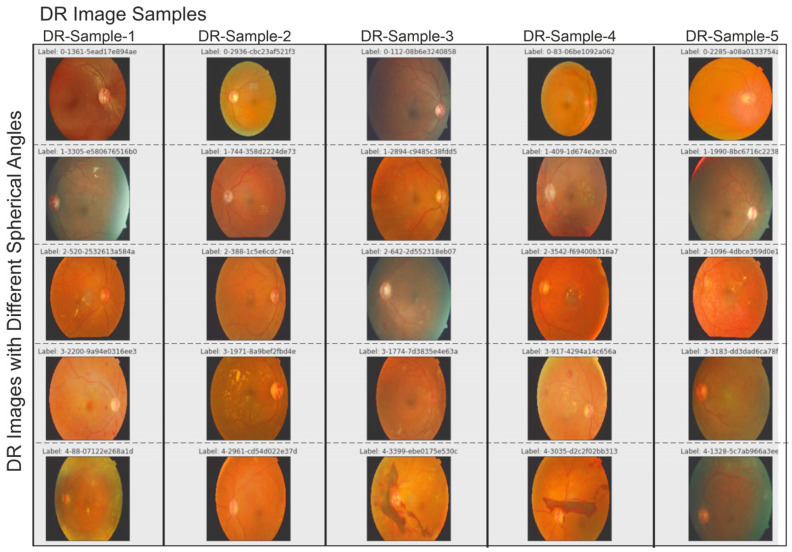
Retrinopatric images used in this study were included in the Kaggle DR dataset. Different color illumination on images was detected before filtering images.

The utilized dataset is a sizable dataset that is openly accessible and was first presented in a Kaggle competition [37]. It comprises four classes of high-resolution images: class 0 (no DR), class 1 (mild DR), class 2 (moderate DR), class 3 (severe DR), and class 4 (all imaging circumstances) (proliferative DR). In Figure 1, a few random samples from various stages of DR in the Kaggle DRD are given. The test and train datasets contain a total of 41,472 and 32,463 images, respectively, given in Figure 1. The test dataset has a comparatively high amount of image samples in each class compared to the imbalanced Kaggle Diabetic Retrinopatric Dataset (DRD). The train–test split used in our trials is the same as that used in the initial competition.

### 3.2. Classification

**Figure 2:**
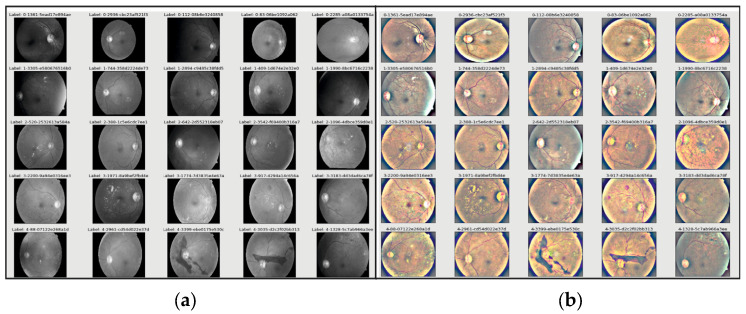
(**a**) Retrinopatric images in grayscale and (**b**) using sigmaX module to clarify DR images with Gauss-based elimination.

The class label is predetermined in the supervised learning method of classification. The model is trained using a training dataset with many images so that it can identify the category or class to which a test sample belongs if it receives any unknown sample images from the test dataset. The anticipated output is compared to the desired outcome to assess how effectively the model is trained. Due to recent improvements in high-performance computing hardware, deep learning is frequently employed in image classification work packages. To determine the severity of DR for a given patient’s retina image, we performed image classification using the sigmaX module to clarify DR images based on Gauss elimination on datasets of varying sizes (Figure 2). For this purpose, sigmaX acts as a coefficient, and in the function, the sample of the color spectrum acts as a power multiplier and is valued as “10” for the most stable resolution to all DR images. The Kaggle DRD was used to test a variety of pre-trained deep-learning CNN architectures.

### 3.3. Applicant Specifications

All the images in the train datasets were subjected to various data augmentation techniques, such as horizontal and vertical shifts, rotation, flipping, and more, to lessen the over-fitting issue. The initial class distributions of samples were unbalanced across all classification datasets. When the pipeline was trained across all batches, the desired distribution was reached so that each class had an equal representation of samples. This was accomplished through the use of the rejection resampling technique, also known as random under-sampling. As a result, while choosing each mini batch for training, the under-sampling approach was used on the move. It was also made sure that the samples were taken at random from the original distribution and shuffled. Regardless of whether sample rejection has been used at the batch level, the training procedure is designed to run for a sufficient number of epochs to cover all training samples. It was noted that each classifier would experience the negative consequences of class imbalance without deploying this rejection resampling technique. However, data augmentation techniques were used in every trial relating to the classification and object-identification challenge. Data augmentation is an approach to regularizing data that helps deep learning models perform better.

### 3.4. Detection of DR Objects Using the CNN Approach

By drawing a bounding box around the object, object detection can be utilized to detect whether an object is present in an image. Whether there are multiple objects in an image determines whether it falls under the single-class object-detection or multi-class object-detection category [27]. The process of recognizing the presence of items in an image and pinpointing their locations in the image is known as object detection and localization. Alzubaidi et al. [4] highlighted the importance of CNN-based deep learning’s widespread application to object identification tasks in various industries, including robotics, surveillance and transportation, autonomous driving, and the medical industry.

**Figure 3:**
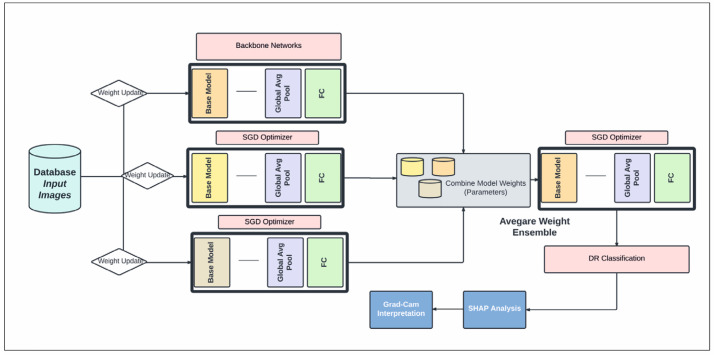
The proposed schematic diagram illustrates our DR diagnosis system, incorporating an average weight ensemble technique utilizing backbone CNN models.

In this study, a CNN architecture is used to extract features from retinal fundus images for the classification of DR into binary and multi-class categories, summarized in Figure 3. Automated approaches must be developed to increase the accuracy of diagnosis and categorize different stages of DR. An approach to reduce computational complexity and improve classification performance is presented in the suggested work. Layer-by-layer abstraction is made feasible by the CNN’s ability to extract symbolic information from the input data through the layer-by-layer stacking of convolution, pooling, and non-linear activation function mapping. The term “feed-forward operation” refers to this procedure. The back-propagation algorithm determines the error by computing the difference between the anticipated and valid values. Traditional deep neural networks face challenges in training as they become deeper, due to issues like vanishing gradients. Residual networks address this by utilizing skip connections or shortcuts (known as identity mappings) to jump over some layers. This allows the network to learn residual functions, making it easier to optimize and train very deep networks effectively. In this study, we used EfficientDet-D0 on the Kaggle DRD that was built on ResNet50.4.3. In addition to that, the segmentation of DR using SHAP was employed. The nomenclature of architectural designations, exemplified by the designation “ResNet50”, conventionally encapsulates the numerical depiction of the total layer count or the hierarchical composition of constituent blocks within the neural network (Figure 3). Precisely, the ResNet50 configuration embodies a convolutional neural network (CNN) architecture featuring 50 layers, predominantly constructed with fundamental structural elements known as residual blocks. These residual blocks are pivotal components facilitating the network’s depth and enabling the successful training of increasingly deeper architectures.

Within the context of variant specifications denoted by appended numerical identifiers, such as “ResNet50.4”, these additional numerals often signify purpose-driven modifications or adaptations intended to tailor the network’s capabilities to specific computational tasks or domain-specific datasets. The appended “.4” in this instance suggests bespoke adjustments engineered to augment the network’s proficiency in targeted applications, such as refined efficacy in object detection, semantic segmentation, or other intricate computer vision tasks. These tailored modifications encompass diverse methodologies, potentially including integrating varied regularization techniques, activation functions, or optimization algorithms strategically implemented to enhance a model’s performance metrics, accelerate convergence rates, or improve generalizability within the designated task domain.

While all these stages were being prepared for publication, Grammarly software played a pivotal role in refining and enhancing the linguistic aspects of the manuscript. The tool was systematically applied to scrutinize the text, providing valuable insights into grammar, syntax, and overall writing quality. This process also contributed to the methodological rigor of the research by ensuring the precise and effective communication of ideas based on AI technology.

## 4. Results

### 4.1. Identifying DR Zones through Enhanced CNN Modeling

**Figure 4:**
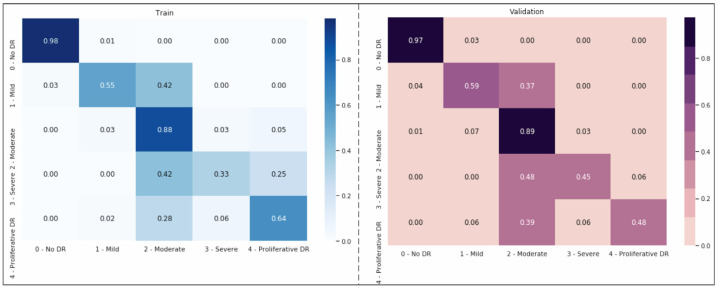
Second-order weighting; Kappa weighting is conducted in the form of LSQ within the matrix set, and the score value obtained here represents the success rate of the modeling.

In this section, we assess our models and contrast the performance of our ensemble model with other configurations based on SHAP schedules by using the optimized DenseNet121 CNN model as our foundation. The input image is separated into distinct regions or segments during image segmentation. The pixels in each area share comparable qualities. To locate items, segmentation is the procedure used to identify boundaries. Each pixel in an image is given a label, with the same label being given to pixels with the same attributes (Figure 4). The foreground and background are divided during segmentation. We can employ numerous pre-trained networks to segment data. We have an integrated encoder and decoder inside the model for CNN designs. Image segmentation brings down the complexity and further simplifies image analysis. It could imply that our model still does not fully understand a crucial “hard exudates” aspect without SHAP design.

Figure 4 shows that the models exhibit a moderate learning tendency throughout the training phase, along with a slightly erratic decrease in validation losses. This behavior indicates that the models exhibit a lack of stability at the end of the training run. The analyzed models show more variance than anticipated and oscillate up and down throughout the training run. Close to the end of the training period, at roughly 10 epochs, we create an ensemble model to address this segmentation problem by averaging the weights from various models (Figure 3). The ensemble weight is then fitted to a clone model from the network, which is anticipated to be a more stable and effective solution. Thus, we opt to employ an exponentially declining average of model weights to evaluate the remaining models.

**Figure 5:**
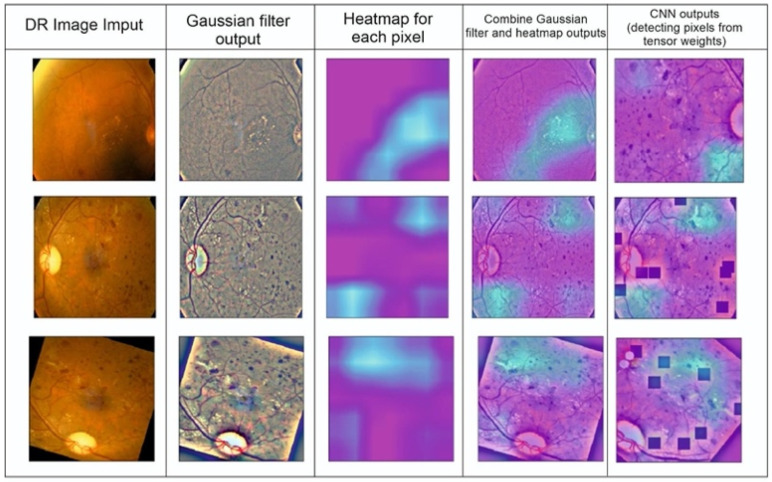
Detection of DR points created by completing CNN segmentation modeling.

We interpreted the model’s segmentation predictions using Grad-CAM and SHAP approaches. The heat map depiction of the few sample fundus images using Grad-CAM for the ensemble model is shown in Figure 5. It includes the original images, as well as heat maps that highlight critical regions with possible DR signs, and a composite of the two. The suggested approach focuses on the afflicted segmentation areas to classify the images with various DR grades. However, this has to be medically validated by thorough studies with a qualified ophthalmologist. The primary takeaway is that we must ensure that our model bases its predictions on correct segmentation data.

### 4.2. Detecting Regions Affected by DR Using SHAP Approach

**Figure 6:**
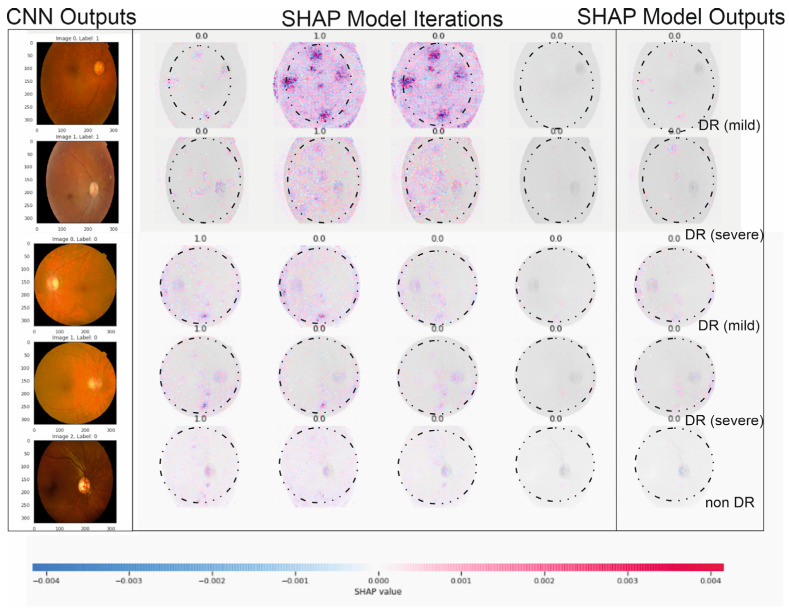
The illustration above describes five outputs in Figure 1 (our five DR levels: 0–5) for extreme values at the edge of SHAP matrixes and random retinopathy images in DR detection zones.

By using SHAP, one can make sure that the model learns relevant features during training and employs the right features for making inferences [47]. Also, in ambiguous situations, the visualization of prominent features might help doctors concentrate on areas of interest where features are most obvious. This not only enhances the transparency of ML models, but also allows practitioners to validate the importance of certain features in medical or diagnostic applications. Understanding the importance of features is crucial, especially in healthcare, where decisions can have a significant impact on patient outcomes. Figure 6 presents an illustration of a SHAP value visualization for one of the ensemble’s models. Red denotes characteristics that increase the output value for a specific DR grade, whereas blue denotes features that decrease the output value for a particular DR grade. The total strength of the characteristics determines the saliency of a topic of interest for a specific DR grade. The efficacy of our deep ensemble model in automatically detecting different DR grades is amply demonstrated by both of these explainability techniques. SHAP [46] allows for the visualization of features that are important for determining the stage of DR illness. The only feasible, consistent, and locally correct additive feature attribution method is SHAP, which combines multiple prior approaches.

Figure 6 showcases the outcomes of employing SHAP analysis to identify key contributing regions within our study’s DR and CNN detection models. Within this illustration, we provide five sample images with a consistent presentation and original image configuration. The SHAP output displayed alongside each image’s right side delineates the specific areas utilized by our deep learning models in making detection predictions. Notably, red pixels signify an increase in the model’s prediction, while blue pixels denote a decrease. Intriguingly, our observation from this figure reveals that the heatmap visualization, outlining critical areas within each fundus image, aligns with suspected regions for both DR and CNN outcomes, demonstrating a more distinct discriminatory ability in the CNN sample. This visualization technique can aid in delineating contributing features by highlighting areas, aiding clinicians in detecting and diagnosing DR by triangulating predictive model results, and annotating SHAP analysis on fundus images. The highlighted areas in the SHAP outputs denote data points influencing the model’s decision making concerning the DR outcome.

The specific example in Figure 6 indicates that the model possessed minimal knowledge about the accurate category, suggesting potential improvements through augmenting the proliferative DR category with additional samples and employing more tailored feature engineering, such as SHAP analysis, in this instance. Additionally, this figure reinforces our prior discussion about the resemblance between moderate- and severe-DR categories (i.e., labels 2 and 3), showcasing similar highlighted areas in both categories, as observed in Figure 5. Furthermore, the outcomes from our SHAP analysis tasks align with a recent study on utilizing deep learning to predict progressive DR in individual patients [23,24,29,41,47], affirming the efficacy of the SHAP technique in interpreting deep learning models for detecting advancing DR.

## 5. Discussion

In this study, we describe a straightforward approach for rating the severity of DR lesions utilizing EfficientNet-B3 and SHAP with image processing. Numerous earlier studies recommending extremely accurate solutions demand extensive machine learning expertise. It is challenging to create the same or better algorithms to duplicate past methods in various situations accurately. Our methods of evaluating DR and removing residuals from lesions were based on a relatively straightforward idea; therefore, if they are put to practical use, the suggested approaches could be replicated in other surroundings.

We carried out straightforward procedures and suggested a simple lesion identification and severity rating scheme for DR. We also offered a numerical approach to blurring image removal. Several earlier researchers manually eliminated fuzzy and subpar images to organize datasets better [24,41]. To improve reproducibility, we developed a numerical threshold identifying a fuzzy retina of 550 × 55 pixel images and created a clean dataset. To the best of our knowledge, our numerical technique for blurry image recognition is the first effort in the field of retinal image analysis to remove hazy and subpar images objectively. The output accuracy was shown to be unaffected by the model type when our EfficientNet-B3 model was compared to other CNN models (Figure 3). This demonstrated that our image preparation and pre-processing techniques were not limited to the EfficientNet-B3 model and could generate results with any dataset meeting predefined requirements.

Using the Kaggle dataset, we trained the EfficientNet-B3 model for DR severity assessment. Only two studies [11,48] that we are aware of have employed EfficientNets for the DR classification task. Using progressive scaling, they attained a quadratic weighted Kappa score of ~0.8 overall and an accuracy of ~0.85 during training. In contrast, our approach, which uses EfficientNet-B3 and RGB visuals, which have a complicated architecture and additional parameters, was more accurate than the first predictions, with a quadratic weighted Kappa score of 0.87 (Figure 4).

**Figure 7:**
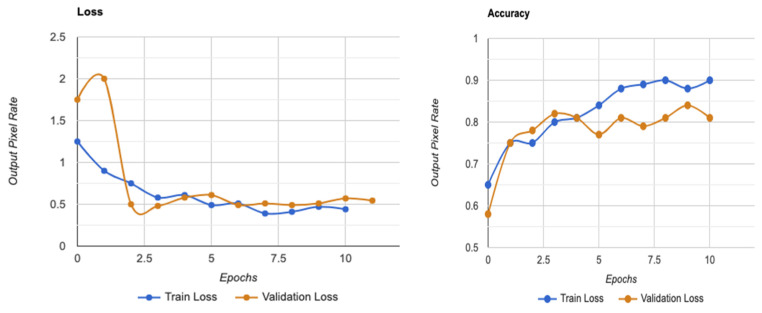
Model loss graph for a trained dataset on utilities in SHAP.

One of the most successful iteration sets was formed by Wang and Yang [49], who used the EfficientNet-B7 model to find referable and vision-threatening DR; Grad-CAM output with finely detailed visualizations demonstrated that their model only recorded DR lesions and did not account for optic discs for prediction. Our methodology demonstrated that EfficientNet, with limited parameters, included all parts of the eye and achieved a high score for DR classification. Moreover, it is seen that the images are determined as DR points as a result of not filtering the DR points before data processing, and the ROC value increases with these apparent images, as shown in the model loss graph of the trained dataset given in Figure 7.

Bodapati et al. [48] employed a deep neural network and a gated attention technique to identify DR using the Kaggle dataset and detect DR points on pre-trained CNN models. One of the most negative handicaps of the method is that spatial pooling techniques are given for obtaining the condensed forms of these representations with data loss. They achieved 97.82% accuracy among more than 3500 total images, and used ~70% for overall training and less than ~20% (about 700 images) for testing. The low inclusion of such data in the system will cause important and detectable DR points to be missed. Similarly, Alyoubi et al. [50] developed a customized CNN model with five convolutional layers and achieved an overall accuracy of 77%. It can be seen that the proposed model performs better than the existing models for the multi-class classification (five classes).

**Figure 8:**
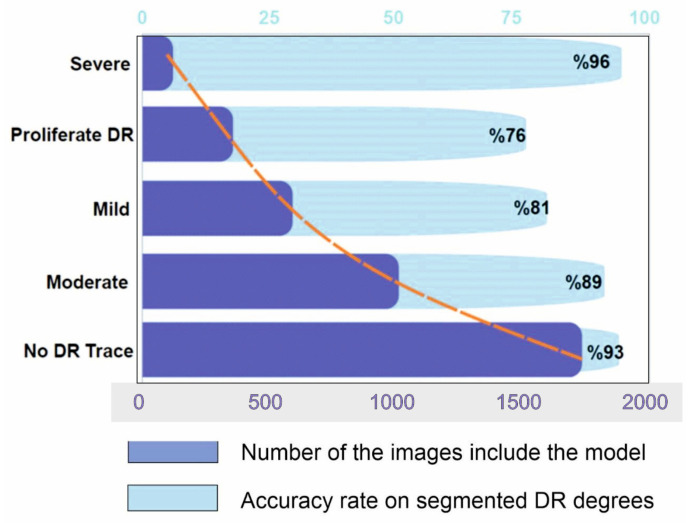
Results of segmentation model from hybrid CNN-SHAP modeling on Kaggle dataset. Dark blue bars represent the number of DR images included in the modeling, whereas light blue bars represent the accuracy rate of segmented DR degrees (the orange line represents the distribution curve of the modeling).

According to different studies (such as Bodapati et al. [48]), a blended multi-modal fusion model has been developed. Some of these studies used grayscale conversion and adaptive histogram equalization, similar to our suggested pre-processing method. They also carried out segmentation by using a matching filter and fuzzy clustering. Unfortunately, they only managed to attain an accuracy of 81%, which is significantly less than the suggested approach. The lower accuracy may be due to the inherent computational constraints of the fuzzy system with matched-filter-based segmentation that cannot ensure optimal ROI localization, especially for fundus images with highly complex architecture and DR features. Undoubtedly, the efficiency of the DR classification is directly related to optimal or accurate ROI identification, feature extraction, and classification. Our suggested approach, on the other hand, aims to apply well-calibrated multilevel enhancement by first enhancing image quality, assuring ideal segmentation, and leading the best possible DR-ROI feature extraction and classification, as seen in Figure 8. As a result, the CNN-based DR approaches outperform the conventional neural network algorithms. Red lesions, including microaneurysms and hemorrhages, are common in DR lesions and can be challenging to identify from thin vessels since they frequently develop close to the vessels and have a similar color. Prior researchers, such as Lazar and Hajdu [51] or Shan and Lee [38], have attempted to differentiate between arteries and red lesions using morphological characteristics; nevertheless, false-negative missed red lesions are still an issue in such a system. We also made an effort to identify vessels using a different method from the original images (Figure 2 and Figure 5); however, accurate blood vessel identification is quite challenging when red lesions are also being sought out for detection. As a result, we did not try to separate red lesions from blood vessels. We encountered the same issue with white lesion detection because the optic disc and contour regions were extracted simultaneously. Further white lesions formed a barrier to the extraction of the optic disc when we attempted to detect the optic disc and exclude the white lesion output. We deduced from these experiments that the future separation of white lesions and optic discs and the separation of red lesions and blood arteries call for some treatments.

Another limitation is our focus on using a single deep learning structure to interpret DR detection through SHAP analysis, which might yield varied outcomes if different models were employed on the same data. Yet, our research’s extensive dataset size and consistent findings essentially diminish this concern. Secondly, due to computational limitations, our deep learning model was set with fewer than 20 training cycles, potentially affecting its learning ability. Nonetheless, the model exhibited high performance in binary classification, and analysis suggests additional training cycles may not significantly alter our study’s conclusions. Thirdly, the relatively small size of the choroidal nevus dataset prompted us to limit training cycles to a maximum of 40 for a more detailed analysis, mitigated by employing feature engineering to enhance predictive abilities despite data size limitations.

There is no known cure for the later stages of DR. The practitioner can examine the patient’s lipid profile, glucose levels, and other risk factors if the diagnosis is made sooner or at a milder stage [38]. After that, enforcing strict control would stop DR from progressing to subsequent stages [1,27,32,33]. The literature for multi-class classification with class-wise metric values is sparse. Precision scores of 93%, 89%, 81%, 76%, and 96%, respectively, for normal, mild, moderate, severe, and DR phases were obtained from Figure 8. In contrast, limited studies [6,20,30,34,42] and review studies ([4,28]) have reached overall scores of 96%, 88%, 75%, 472%, and 95% for the recall metric for normal, mild, moderate, severe, and PDR phases, respectively.

As can be observed, the model outperformed the current method regarding precision and recall scores for the earlier stages, particularly for the normal, mild, and moderate stages. As a result, the suggested model performed better than existing techniques regarding overall accuracy, class-wise precision, and recall values, and achieved an overall high value of AUC for both binary and multi-class classifications [17]. This was especially true for DR detection in the early stages. With this knowledge, medical professionals can create a strategy for adequately managing diabetes and perhaps avert the later development of DR, which can cause vision loss.

## 6. Conclusions

The diagnosis of diabetic retinopathy (DR) and non-DR retinal images was suggested in this research using a deep learning-modified CNN algorithm. Modifying a pre-trained CNN network makes sense to skip the lengthy convolutional system training procedure. According to the experimental findings, detection accuracy can be improved by fusing derived frequency domain characteristics with spatial features. The suggested approach produced either two- or three-dimensional feature maps, and the two-pathway design outperformed individual paths by a wide margin. Although the results of our numerous simulations indicate that the proposed methodology is sensitive to training and testing sizes, it is not sensitive to the training and testing sets that were chosen as unsystematic. This study also classifies CNN data using the SHAP algorithm with appropriate kernel functions. Different kernel settings and classifiers were compared in this comparative study. The objective of the comparison study was to show how different kernel function levels affected the accuracy of diagnosing cases of DR in screening.

By utilizing the suggested kernel functions, the proposed technique can handle the increased degree of dimensionality of the CNN output data and produce acceptable results. The proposed structure is anticipated to be further improved by the inclusion of some retinal image features along with the CNN feature outputs in this work, and more research is advised. In this study, we showcased the effectiveness of the EfficientNet model in accurately detecting lesions associated with DR and categorizing its severity grades using basic image-processing techniques. Our findings highlight the potential of these simplified methods for detecting DR lesions and grading their severity, paving the way for the development of user-friendly computer-aided diagnostic systems.

This study focuses on demonstrating the capabilities of the EfficientNet model and prompts recommendations for future research. Specifically, it suggests exploring more accessible techniques that leverage the EfficientNet model for diagnosing DR. By employing a CNN architecture and a straightforward image-processing approach using SHAP iterations, our model effectively grasped the nuances of DR severity grading and associated distinctions, even with a limited open-source dataset. Thus, this study highlights the necessity for more exploration into accessible techniques utilizing the potential of the EfficientNet model for diagnosing DR. We recognize the study’s constraints and encourage ongoing exploration in this domain.

## Data Availability

The data presented in this study are openly available in Kaggle Diabetic Retinopathy [47]. This dataset is publicly available.
